# Real-Time Biomarkers of Liver Graft Quality in Hypothermic Oxygenated Machine Perfusion

**DOI:** 10.3390/jcm14020471

**Published:** 2025-01-13

**Authors:** Andriy Zhylko, Marcin Morawski, Paweł Rykowski, Maciej Krasnodębski, Anya Wyporski, Jan Borkowski, Dmytro Zhylko, Konrad Kobryń, Rafał Stankiewicz, Jan Stypułkowski, Wacław Hołówko, Waldemar Patkowski, Tadeusz Wróblewski, Benedykt Szczepankiewicz, Barbara Górnicka, Magdalena Mielczarek-Puta, Marta Struga, Marek Krawczyk, Michał Grąt

**Affiliations:** 1 Department of General, Transplant and Liver Surgery, Medical University of Warsaw, Banacha 1A, 02-097 Warsaw, Poland; marcin.morawski@wum.edu.pl (M.M.); marek.krawczyk@wum.edu.pl (M.K.); michal.grat@wum.edu.pl (M.G.); 2 Doctoral School, Medical University of Warsaw, 02-091 Warsaw, Poland; 3 Computer Engineering Division, New York University Abu Dhabi, Abu Dhabi P.O. Box 129188, United Arab Emirates; 4 Department of Pathology, Medical University of Warsaw, 02-004 Warsaw, Poland; 5 Department of Biochemistry, Medical University of Warsaw, 02-097 Warsaw, Poland

**Keywords:** allograft function, biomarkers, liver transplantation, machine perfusion

## Abstract

**Background**: Hypothermic oxygenated machine perfusion has emerged as a strategy to alleviate ischemic-reperfusion injury in liver grafts. Nevertheless, there is limited data on the effectiveness of hypothermic liver perfusion in evaluating organ quality. This study aimed to introduce a readily accessible real-time predictive biomarker measured in machine perfusate for post-transplant liver graft function. **Methods**: The study evaluated perfusate analytes over a 90-day postoperative period in 26 patients randomly assigned to receive a liver graft following dual hypothermic machine perfusion in a prospective randomized controlled trial. Machine perfusion was consistently conducted end-ischemically for at least 120 min, with real-time perfusate assessment at 30-min intervals. Graft functionality was assessed using established metrics, including Early Allograft Dysfunction (EAD). **Results**: Perfusate lactate concentration after 120 min of machine perfusion demonstrated significant predictive value for EAD (AUC ROC: 0.841, *p* = 0.009). Additionally, it correlated with post-transplant peak transaminase levels and extended hospital stays. Subgroup analysis revealed significantly higher lactate accumulation in livers with post-transplant EAD. **Conclusions**: Liver graft quality can be effectively assessed during hypothermic machine perfusion using simple perfusate lactate measurements. The reliability and accessibility of this evaluation support its potential integration into diverse transplant centers.

## 1. Introduction

Liver transplantation is the primary curative option for various diseases, with a growing list of indications. However, the persistent shortage of available organs remains a significant obstacle to meeting the increasing demand. To address this issue, the utilization of extended criteria donors (ECD) has emerged as a potential solution to expand the pool of organs for transplantation [1]. Despite their promise, ECD liver grafts are highly susceptible to ischemic-reperfusion injury (IRI), leading to a higher incidence of post-transplant complications [2,3,4].

Traditionally, transplant surgeons have relied on pre-procurement donor demographic data, laboratory findings, and macroscopic liver appearance to determine the suitability of liver grafts for transplantation. However, these methods are often subjective and may fail to fully capture the functional viability of the graft. Clinicians face significant challenges in balancing the demand for donor organs with the risk of graft failure or recipient complications, particularly when dealing with marginal or high-risk grafts. Although multivariable prognostic models [5,6,7,8], like the Donor Risk Index (DRI), have been developed to reduce uncertainty, the quest for more accurate assessment methods is ongoing.

The perspective on organ quality assessment has significantly shifted with the widespread adoption of machine perfusion techniques. Extensive efforts have been focused on establishing optimal validation criteria for effectively assessing liver quality during normothermic machine perfusion (NMP) [9,10,11,12,13,14,15]. While NMP provides valuable insights into how well a liver handles accumulated injuries, it is important to note that it may also contribute to organ damage by promoting the production of reactive oxygen species (ROS) during normothermic reperfusion following IRI [16,17].

An alternative approach is hypothermic oxygenated machine perfusion (HOPE), which promotes slow activation of mitochondrial metabolism under oxygenated perfusion at low temperatures [16]. This controlled metabolism of ischemically accumulated succinate with adenosine triphosphate (ATP) replenishment [18,19] minimizes ROS generation [16,20,21] and mitigates IRI, leading to improved long-term outcomes for ECD grafts [19,22,23,24,25,26,27,28,29]. However, assessing liver function during the HOPE procedure presents challenges due to temperature-dependent metabolic effects.

Recent research by Muller et al. [30] proposes measuring perfusate flavin mononucleotide (FMN) concentration as a potential biomarker to predict liver function after transplantation. Although data on this approach are limited, primarily originating from the transplant center in Zurich, it holds promise for improving organ quality assessment. The scarcity of data may be partly attributed to the specialized equipment needed for performing these measurements.

The primary aim of this study is to introduce a novel predictive biomarker for post-transplant liver graft function. Specifically, we propose a real-time analysis of lactate concentration in perfusate samples—a widely available, routinely used, and cost-effective approach. This biomarker is intended to offer a dependable and easily accessible method for predicting the performance of liver grafts after transplantation.

## 2. Materials and Methods

### 2.1. Study Cohort

This study analyzed a cohort of 26 patients who underwent liver transplantation from donors after brain death (DBD) within a single-center, prospective, randomized controlled trial (NCT04812054), conducted between April 2021 and May 2022 [29]. All participants were selected based on national transplant policy regulations and met the inclusion criteria: age above 18 years and provision of informed consent before enrollment. Patients were randomly assigned to one of two groups: 26 patients received livers treated with dual hypothermic oxygenated machine perfusion (dHOPE), while 76 patients received conventional static cold storage (SCS). The trial aimed to evaluate whether routine hypothermic machine perfusion is necessary for liver grafts that were already accepted for transplantation following standard cold storage. Details of the trial outcomes and its primary analysis have been published elsewhere [29,31].

### 2.2. Procurement, Machine Perfusion, and Graft Implantation

Liver grafts were procured following the standard procurement protocol, including a cold in situ flush using Institut Georges Lopez-1 (IGL-1) solution (StoreProtect Plus, Carnamedica, Warsaw, Poland). Only liver grafts from DBD donors were included in the study. Upon arrival at the transplant center, livers underwent final assessments to determine suitability for transplantation. During bench preparation, each liver was flushed with 1 L of cold IGL-1 solution to remove residual blood and debris.

Machine perfusion was performed end-ischemically using the Liver Assist device (XVIVO, Gothenburg, Sweden). Grafts allocated to dHOPE underwent perfusion for a minimum of 120 min. Perfusion parameters were standardized: portal vein pressure was maintained at 3–5 mmHg, arterial pressure was capped at 25 mmHg, and the temperature was consistently set at 12 °C. If the recipient hepatectomy extended beyond the initial 120 min of dHOPE, perfusion continued until the grafts were required for implantation. At the time of transfer, grafts were disconnected from the perfusion device and directly implanted. Perfusion was conducted using 2 L of oxygenated (≥450 mmHg) University of Wisconsin (UW) Machine Perfusion Solution (PumpProtect, Carnamedica, Warsaw, Poland) and the perfusate was not replaced during the procedure.

Implantation methods included either the piggy-back or classical technique, based on the surgeon’s discretion and clinical circumstances. The reperfusion sequence followed standard practice, starting with portal vein reperfusion and subsequently followed by arterial reperfusion.

### 2.3. Perfusate Analysis During dHOPE

Perfusate samples were collected every 30 min during dHOPE to monitor specific parameters. Lactate and oxygen levels were analyzed using the Radiometer ABL800 Flex device (Radiometer Medical ApS, Copenhagen, Denmark). FMN was quantified using the FS5 Spectrofluorometer (Edinburgh Instruments, Livingston, UK). The FMN analysis protocol involved excitation with monochromatic light at 450 nm, with emission measured between 500 and 600 nm [30]. Measurements were obtained with a 1 nm step size, a dwell time of 0.250 s, and three repeated scans per sample to ensure accuracy and reproducibility.

### 2.4. Data Collection and Statistical Analysis

Comprehensive data collection was performed prospectively, encompassing donor and recipient demographics, perioperative variables, perfusate analyses, post-transplant laboratory results, and clinical outcomes. Donor liver grafts were evaluated using the donor risk index (DRI) and quantified steatosis through routine wedge biopsies post-reperfusion. Graft functionality post-transplantation was assessed using metrics including Early Allograft Dysfunction (EAD) [32], Model for Early Allograft Function (MEAF) [33], and Liver Graft Assessment Following Transplantation (L-GrAFT7) [34] scores. Post-transplant outcomes over 90 days were analyzed to identify correlations between perfusate biomarkers and clinical outcomes.

Statistical analyses were conducted using GraphPad Prism 9. Continuous variables were presented as median values along with the interquartile range (IQR) and compared using the two-sided Mann-Whitney test. Categorical variables were assessed using the two-sided Fisher’s exact test and expressed as percentages. Correlation analyses were computed using Spearman’s rank correlation coefficient (rs). The sensitivity and specificity of the selected parameters’ predictive value were evaluated through receiver-operating characteristic curve (ROC) analysis. The Youden index was employed to identify the cut-off value associated with the highest specificity and sensitivity. The corrected Akaike Information Criterion (cAIC) was used to compare multiple logistic regression models. Statistical significance was defined at a two-sided *p*-value of <0.05.

## 3. Results

### 3.1. Donor and Recipient Characteristics

A total of 26 patients, with a median age of 46 (39–62) years, underwent liver transplantation using a DBD liver following the dHOPE procedure. The majority of patients, comprising 18 out of 26 (69.2%) cases, were males. Regarding the severity of liver disease, the median MELD score was 12.0 (8.0–21.0), and 15 out of 26 (57.7%) patients were classified under Child-Turcotte-Pugh class A. The primary indications for transplantation were alcohol-related liver disease or primary sclerosing cholangitis, as indicated in Table 1.

Concerning donors, their median age was 53 (40–60) years, and the DRI was 1.7 (1.4–2.0). Only 1 out of 26 (3.8%) livers exhibited large droplet histopathological steatosis levels exceeding 30% (Table 2).

### 3.2. Machine Perfusion Characteristics

Following a median cold ischemic time (CIT) of 450 min (IQR ranging from 420 to 550 min), the livers underwent dHOPE perfusion for at least 120 min. In cases where hepatectomy was still ongoing, the perfusion time was extended, resulting in a median machine perfusion time of 120 (120–180) min. The total hypothermic time, including both CIT and machine perfusion time, had a median value of 608 (560–687) min (Table 3).

### 3.3. Prediction of Graft Function

**Figure 1:**
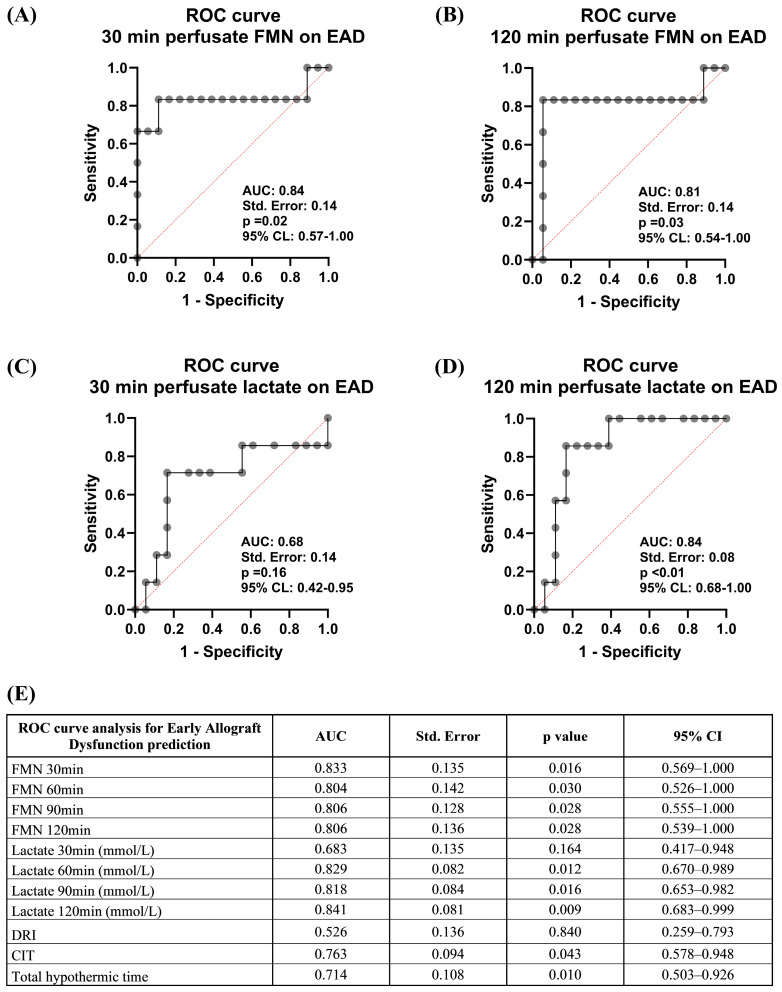
Predicting early allograft dysfunction. (**A**,**B**) ROC curve analysis of perfusate FMN measurements after 30 and 120 min of perfusion for EAD prediction. (**C**,**D**) ROC analysis of perfusate lactate measurements after 30 and 120 min for predicting EAD. (**E**) Overview of ROC analysis outcomes for perfusate FMN and lactate measurements at different time points, DRI, CIT, and total hypothermic time concerning EAD predictability. ROC, receiver operating characteristic; AUC, area under the curve; CI, confidence interval; FMN, flavin mononucleotide; EAD, early allograft dysfunction; DRI, donor risk index; CIT, cold ischemic time.

Consistent with previous reports, the analysis of FMN after 30 min of machine perfusion showed predictive value for EAD [c statistic 0.833, 95% confidence interval (CI) 0.569–1.000, *p* = 0.016] (Figure 1 and Figures S1–S3). Notably, the predictive ability of perfusate lactate concentration for EAD was observed only during prolonged dHOPE. The area under the ROC curve (AUC) stood at 0.683 (95% CI 0.417–0.948, *p* = 0.164) after 30 min of perfusion and improved to 0.841 (95% CI 0.683–0.999, *p* = 0.009) after 120 min of perfusion. Additionally, there was a significant correlation between perfusate FMN measured after 30 min of perfusion and lactate levels measured after 120 min of dHOPE (Figure S4).

**Figure 2:**
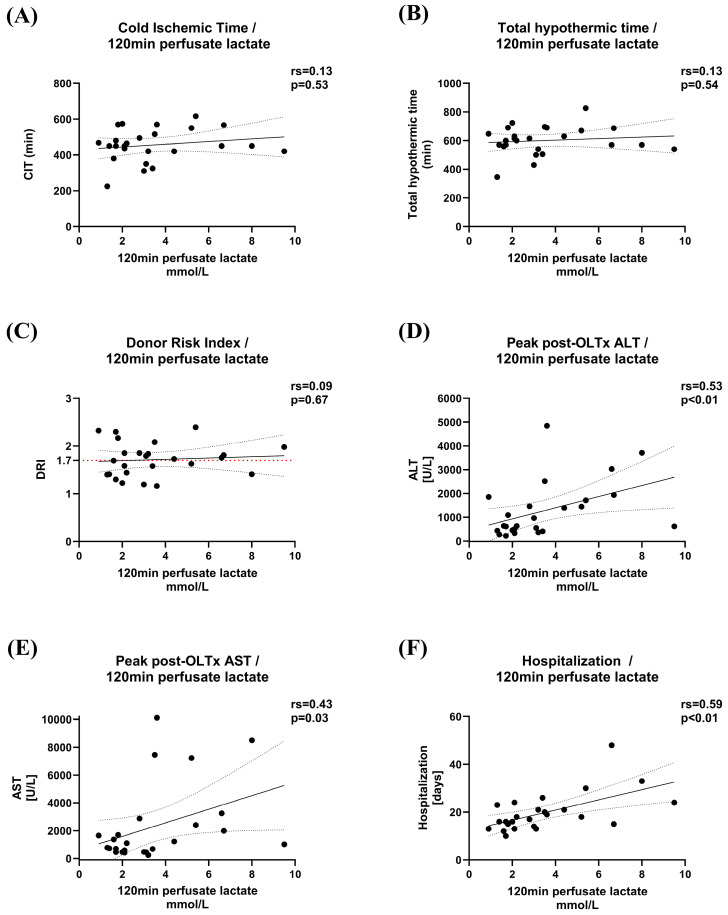
Relationship between perfusate lactate and liver graft characteristics, as well as post-transplant outcomes. There was no notable correlation between liver graft characteristics, including cold ischemic time (**A**), total hypothermic time (**B**), and donor risk index (**C**), with perfusate lactate concentrations measured after 120 min of perfusion. However, perfusate lactate levels demonstrated a significant correlation with post-transplant biochemical laboratory parameters associated with liver injury, such as peak ALT (**D**) and AST (**E**), along with the duration of hospitalization (**F**). OLTx, orthotopic liver transplantation; ALT, alanine aminotransferase; AST, aspartate aminotransferase.

Furthermore, the concentration of perfusate lactate after 120 min of dHOPE was found to be unrelated to CIT, total hypothermic time, and DRI, whereas it exhibited significant correlations with peak transaminase levels after liver transplantation and prolonged hospital stay (Figure 2).

### 3.4. Prediction of Post-Transplant Complications

**Figure 3:**
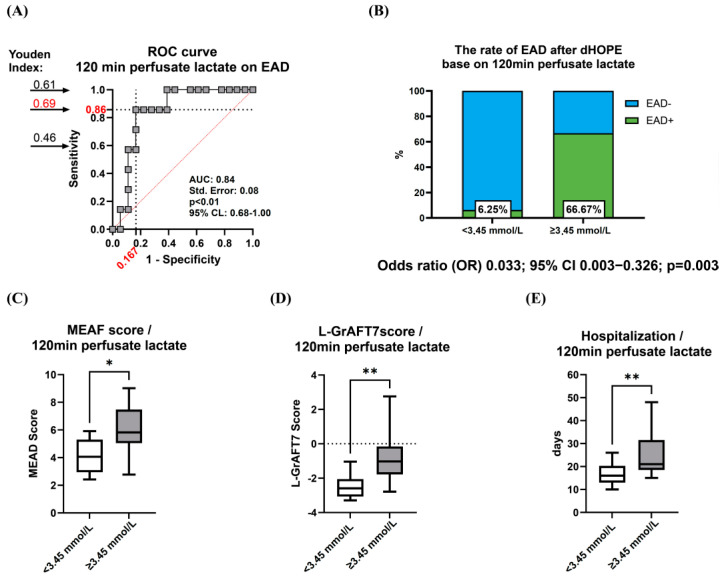
Identification of ’Increased-Risk’ liver grafts based on perfusate lactate concentration. (**A**) Determining the cut-off value for perfusate lactate level using the Youden index to classify ‘increased-risk’ liver grafts. (**B**) Liver grafts with perfusate lactate concentration exceeding 3.45 mmol/L after 120 min of machine perfusion exhibited a higher incidence rate of EAD. (**C**) Recipients of ‘increased-risk’ liver grafts experienced notably extended hospital stays. Liver grafts exhibiting perfusate lactate concentration exceeding 3.45 mmol/L after 120 min of perfusion displayed notably compromised post-transplant liver function, as indicated by MEAF (**D**) and L-GrAFT7 (**E**) scores. * Indicates a statistically significant difference *p* < 0.05. ** *p* < 0.01. ROC, receiver operating characteristic; AUC, area under the curve; EAD, early allograft dysfunction; CI, confidence interval; MEAF, Model for Early Allograft Function; L-GrAFT, liver graft assessment following transplantation.

Consequently, livers undergoing dHOPE perfusion were divided into two risk groups, established according to the perfusate lactate concentrations measured after 120 min of perfusion. The threshold for the most accurate prediction of EAD was determined to be 3.45 mmol/L of perfusate lactate. This value was subsequently employed as the cut-off point to characterize ‘increased-risk’ liver grafts (Figure 3A).

The incidence of EAD after dHOPE, categorized by perfusate lactate concentration, was 6% for cases with low lactate levels and 67% for those with high lactate levels (Figure 3B). The evaluation of ‘increased-risk’ liver grafts’ function post-transplantation demonstrated higher MEAF scores [5.82 (5.05–7.47) vs. 4.06 (2.95–5.30)] (Figure 3D) and L-GrAFT7 scores [−1.02 (−1.78–−0.16) vs. −2.59 (−3.07–−2.06)] (Figure 3D), leading to a significantly prolonged hospital stay [21.0 (18.5–31.5) vs. 16.0 (13.0–20.3)] in comparison to dHOPE-perfused livers with perfusate lactate levels below 3.45 mmol/L after 120 min of perfusion (Figure 3E).

Additionally, patients who encountered EAD exhibited a greater frequency of 90-day Clavien-Dindo [36] grade 3 or higher complications [71.4% vs. 15.8%, odds ratio (OR) 13.3, 95% CI 1.94–79.84, *p* = 0.014], a notably extended stays in the intensive care unit (ICU) [7 (5–13) days vs. 5 (4–5) days], and higher 90-day comprehensive complication index (CCI) [37] [39.7 (8.7–58.1) vs. 20.9 (0.0–22.6)] (Figure S5). Furthermore, 3 out of 7 patients who experienced EAD were diagnosed with anastomotic biliary strictures, with 2 of these 3 requiring endoscopic intervention, compared to 0 out of 18 patients without EAD [42.9% vs. 0%, *p* = 0.02] during the 1-year follow-up.

Upon analyzing donor and recipient characteristics between the risk groups, no significant differences were observed, except for liver graft weights, as indicated in (Table 4 and Table S1). The assessment of IRI severity [38] in post-reperfusion biopsies did not reveal any significant differences between the risk groups, with no-to-mild IRI changes observed in the majority of patients (19 out of 26, or 76%). Remarkably, liver grafts with a perfusate lactate concentration exceeding 3.45 mmol/L after 120 min of dHOPE exhibited a greater weight of 1843 (1800–2350) compared to 1737 (1400–1811), *p* = 0.008. Meanwhile, a significant correlation between graft weight and perfusate lactate concentration was observed as early as 30 min into perfusion, and this correlation persisted consistently throughout the entire 120-min perfusion period (Figure S6).

While no significant differences in liver weight emerged when assessing donor and recipient characteristics in relation to EAD occurrence, liver grafts experiencing post-transplant EAD exhibited a significantly longer CIT [516 (450–570) vs. 450 (380–480) min, *p* = 0.041] (Table 5 and Table S2).

**Figure 4:**
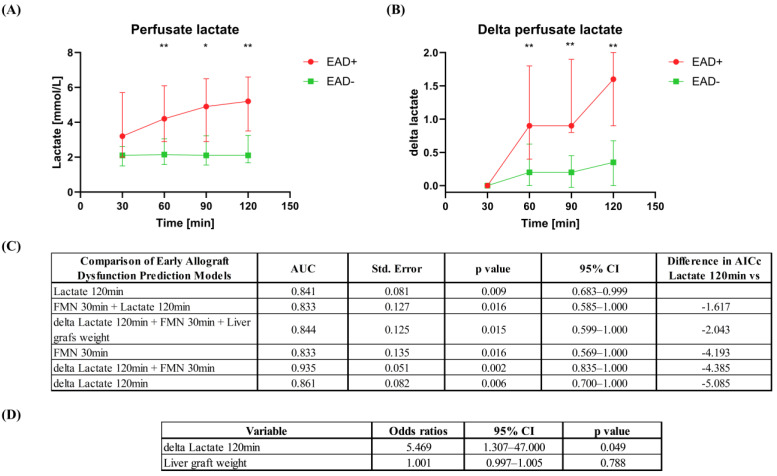
Perfusate lactate accumulation pattern during dHOPE. (**A**,**B**) Liver grafts with post-transplant EAD exhibited a notably higher accumulation of lactate in the perfusate throughout the duration of dHOPE. The delta perfusate lactate was determined as the difference in lactate concentration after 30 min of perfusion and at subsequent time points. (**C**) Summary of ROC analysis for EAD prediction and the difference in AICc between univariable and multivariable prediction models. The difference in AICc was calculated by subtracting the AICc of the univariable prediction model based on perfusate lactate concentrations after 120 min of perfusion from the AICc of subsequent prediction models. (**D**) Multivariable analysis using logistic regression for EAD prediction based on delta lactate levels, while controlling for liver graft weight. * Indicates a statistically significant difference *p* < 0.05. ** *p* < 0.01. ROC, receiver operating characteristic; AUC, area under the curve; CI, confidence interval; FMN, flavin mononucleotide; EAD, early allograft dysfunction; AICc, Akaike Information Criterion corrected.

Upon evaluating perfusate lactate concentrations during dHOPE, a significant observation emerged: livers encountering post-transplant EAD accumulated markedly higher levels of lactate during perfusion (Figure 4A,B). Additionally, the change in lactate concentration over 120 min of perfusion, measured as delta lactate (the difference between lactate concentrations at 30 min and 120 min), showed promise as a parameter for EAD prediction models. A univariate logistic regression model for predicting EAD based on delta lactate exhibited a notably lower corrected Akaike Information Criterion (cAIC) value of 24.62, compared to a cAIC value of 29.71 for the model using the absolute perfusate lactate concentration after 120 min of perfusion (Figure 4C). Furthermore, in multivariable analysis, delta lactate over 120 min was a significant predictor of EAD with an OR of 5.469 (95% CI 1.307–47.000, *p* < 0.05) when controlling for liver graft weight (Figure 4D).

## 4. Discussion

The need for a real-time, cost-effective, and reproducible method for liver assessment remains critical to enhancing the utilization of high-risk grafts, particularly those from donors after cardiac death (DCD), which currently have a utilization rate of approximately 25% [39]. Although various biomarkers, such as high-resolution respirometry, real-time confocal microscopy [40], metabolomic biosignatures [41,42], and lipid fingerprints [43,44], have been identified through intricate laboratory analyses, their translation into clinical practice remains limited due to the complexity and resource-intensive nature of these techniques. Additionally, most prediction models for EAD based on these biomarkers were developed using mixed populations of DCD and DBD donors, despite significant differences in biomarker levels between these donor types. Such differences could affect the efficacy of the reported models and highlight the potential advantages of donor-type-specific calculations.

IRI remains the Achilles’ heel of liver transplantation, contributing to graft dysfunction and postoperative complications. During the ischemic phase, the lack of oxygen causes fumarate to serve as a terminal electron acceptor in the mitochondria respiratory chain, leading to succinate accumulation [45]. Upon reperfusion, the rapid re-oxidation of accumulated succinate triggers reverse electron transport at mitochondrial complex I, resulting in extensive ROS generation and the dissociation of FMN from the complex [16,46,47].

The assessment of FMN concentration in perfusate during hypothermic oxygenated machine perfusion of liver grafts has demonstrated robust predictability for post-transplant outcomes, albeit requiring a spectrofluorometric device for analysis [30,48]. Moreover, the widespread clinical standardization of these measurements might be affected by variations in the optical components of different commercially available spectrofluorometers.

In this context, we propose the use of the widely available and commonly used lactate measurement as an innovative predictive biomarker for assessing liver graft quality during hypothermic oxygenated machine perfusion. Unlike fluorescent markers, lactate can be readily quantified using a standard blood gas analyzer, making it a versatile tool suitable for implementation across various transplant centers.

In line with previous findings [30,48], we observed that liver graft quality assessment can be conducted within the first 30 min of the HOPE procedure by analyzing FMN concentration in the perfusate. The release of FMN serves as an indicator of mitochondrial damage, acting as the initial “domino” in triggering a cascade of cellular metabolic dysregulation [49]. Therefore, evaluating organ function through perfusate lactate concentration requires a prolonged HOPE procedure. The lactate concentration measured in perfusate samples after 120 min of HOPE emerged as a robust predictor of post-transplant EAD and clinically significant outcomes, including ICU and hospital stays or cumulative complications. Furthermore, grafts experiencing EAD demonstrated significantly higher lactate accumulation levels during perfusion compared to livers with favorable post-transplant functions.

EAD, as defined by Olthof et al. [32], is a well-recognized early clinical indicator that provides a measurable endpoint for assessing liver graft function. Studies have shown that EAD is associated with higher risks of graft failure, biliary complications, and reduced long-term survival. Although more robust models have been developed in recent years, EAD remains widely used as an endpoint in studies aimed at developing predictive biomarkers for pre-implantation organ assessment during hypothermic machine perfusion. For these reasons, we chose EAD as an endpoint in our study. Consistent with previous reports, we observed a higher incidence of both early and late-onset post-OLTx complications in patients who experienced EAD.

Analysis of patients who experienced EAD revealed a significant extension in CIT duration compared to liver grafts without post-transplant EAD. However, CIT duration alone was insufficient to predict EAD or perfusate lactate concentrations after 120 min of perfusion. The duration of hypoxia has been shown to correlate with ischemically accumulated succinate and subsequent ROS production upon reperfusion, contributing to increased liver damage. Thus, although not the primary instigator of liver damage, prolonged CIT in livers with EAD appears to be a contributing factor to that extent of injury.

Several studies have defined a minimum HOPE procedure duration of 1 h [30,45], providing sufficient organ evaluation based on FMN analysis [30,48]. However, a one-hour perfusion may be inadequate for severely macrosteatotic grafts [50]. In contrast, extended HOPE perfusion, even up to 20 h, has been proven to be safe and effective [51]. Therefore, a minimum 2-h HOPE duration is reasonable to fully leverage its benefits, streamline logistics [52], and gain deeper insights into the liver’s metabolic function, particularly regarding total lactate concentration and accumulation patterns.

Lactate clearance assessment is commonly used during NMP to evaluate liver graft viability. Under physiological conditions, hepatocytes in the periportal zone of the hepatic lobule metabolize lactate, making reduced clearance a robust indicator of pan-lobular injury [53]. However, lactate metabolism appears to be inhibited at low temperatures, as seen in hypothermic perfusion of liver grafts with excellent post-transplant outcomes, where significant lactate clearance was not observed. This finding underscores the altered metabolic processes at lower temperatures and highlights the need for specific quality assessment biomarkers tailored to the HOPE procedure.

Moreover, the type of HOPE perfusion—whether single portal perfusion or dual perfusion via both the portal vein and hepatic artery—may impact metabolic pathways, adding complexity to univariate data analysis. Although we performed dual HOPE, recent preclinical [54] and real-world clinical data [55] have shown excellent long-term outcomes for HOPE-treated organs, regardless of perfusion type or device. This supports the view of underlying metabolic pathway similarity.

Metabolomic profiling of back-table liver graft biopsies using high-resolution magic-angle-spinning nuclear magnetic resonance revealed elevated lactate levels in livers with post-transplant EAD, showing a robust ROC AUC of 0.906 (95% CI 0.813–0.999) [56]. However, the weight of liver grafts may significantly impact perfusate lactate concentrations [57], contrasting with directly studying liver samples. In our study cohort, liver grafts with lactate concentrations of ≥3.45 mmol/L after 120 min of perfusion were significantly heavier. While there was a significant correlation between liver weight and the 120-min perfusate lactate concentration, larger grafts may release greater quantities of biomarkers into a fixed volume of perfusate, independent of graft quality. This is supported by the correlation between weight and lactate concentration, which was observed from the start of perfusion and persisted consistently, albeit with a decreasing trend. Additionally, the absence of weight differences when comparing the occurrence of EAD further supports this hypothesis. This observation might explain why about one-third of livers with perfusate lactate concentrations exceeding 3.45 mmol/L displayed excellent post-transplant function. Furthermore, when comparing lactate concentration trends during dHOPE, livers with post-transplant EAD demonstrated significantly higher lactate accumulation. This underscores the importance of assessing accumulation patterns rather than solely relying on weight-dependent total lactate concentrations to predict graft dysfunction accurately in future studies.

We hypothesize that lactate accumulation during the dHOPE procedure results from mitochondrial damage and an associated increase in anaerobic metabolism. A significant correlation was found between FMN, a marker of mitochondrial damage, and lactate levels in the perfusate after 30 and 120 min of dHOPE. Given their comparable predictive abilities for EAD, these biomarkers, although measured at different times, may partially reflect the same pathological process in liver grafts. Furthermore, the absence of exacerbated IRI changes in liver grafts with elevated lactate levels in the perfusate supports this speculation.

In clinical practice, HOPE is typically performed end-ischemically, starting shortly before or at the time of the recipient’s diseased liver explantation. In this context, the assessment of 120-min perfusate lactate levels may not influence graft use, as the transplant process is already at an irreversible stage. However, advancements in prolonged liver perfusion techniques and reports from the first-in-human clinical trial [58] demonstrating the safety and feasibility of HOPE for extending preservation time to enable daytime transplantation support further validation of perfusate lactate levels as a potential decision-aiding biomarker for liver quality.

This study underscores the need for further validation of perfusate lactate levels as a predictive biomarker in larger, multicenter cohorts, encompassing diverse donor types and graft risk profiles. Future research should also investigate combining lactate with other biomarkers, such as FMN, to create a comprehensive panel for real-time graft evaluation during perfusion.

Advancements in perfusion technology, including extended HOPE protocols and AI-driven monitoring systems, provide opportunities to enhance predictive accuracy and integrate biomarker assessment into clinical workflows. Furthermore, validating lactate-based biomarkers in the context of prolonged perfusion protocols could support the feasibility of daytime transplantations and improve graft utilization.

The modulation of the gut-liver axis through fecal microbiota transplantation (FMT) has shown promise in liver cirrhosis by improving gut barrier integrity, reducing systemic inflammation, and mitigating complications such as hepatic encephalopathy and infections [59]. These benefits may also hold potential for post-transplant outcomes by reducing systemic inflammation and supporting recovery in advanced liver disease. Preliminary studies have demonstrated that FMT can favorably alter the gut microbiome and improve clinical outcomes in cirrhotic patients, suggesting its potential utility in transplantation settings. Future research should evaluate the integration of FMT with advanced perfusion strategies to enhance graft quality and long-term outcomes in liver transplantation.

## 5. Conclusions

The study highlights the effectiveness of simple lactate measurement for assessing the quality of liver grafts during HOPE. However, several limitations should be considered. The relatively small sample size included only a few liver grafts with steatosis levels exceeding 30%, and the donor pool was limited to DBD donors, as DCD donors in Category III of the Maastricht classification are not considered acceptable in Poland. Additionally, all perfused livers were accepted for transplantation prior to dHOPE perfusion, with most recipients presenting low Child-Turcotte-Pugh class scores and achieving a 90-day survival rate of 96%. These factors suggest that the severity of post-transplant complications could be greater in high-risk recipients and marginal liver grafts with elevated perfusate lactate concentrations.

In conclusion, these findings support the potential of perfusate lactate concentration measurement during HOPE as a reliable method for evaluating liver graft function. This approach could reduce the need for more costly NMP evaluations in specific cases and improve liver graft allocation by balancing risks associated with recipient and graft complications.

## Figures and Tables

**Table 1:** Recipient characteristics.

Recipient Characteristics	dHOPE (n = 26)
Age in years (range)	46 (39–62)
Sex—n (%)	
Female	8 (30.8)
Male	18 (69.2)
Body mass index (kg/m^2^)	26 (23–28)
Childe-Turcotte-Pugh class—n (%)	
A	15 (57.7)
B	9 (34.6)
C	2 (7.7)
Model for end-stage liver disease score	12.0 (8.0–21.0)
ALT at OLTx (IU/mL)	63 (21–132)
AST at OLTx (IU/mL)	61 (32–111)
Albumin at OLTx (g/dL)	3.9 (3.5–4.7)
Bilirubin at OLTx (mg/dL)	1.6 (0.8–9.3)
INR	1.2 (1.0–1.3)
Creatinine (mg/dL)	0.94 (0.73–1.20)
Transplant indication—n (%)	
Alcohol-related liver disease	6 (23.1)
Primary sclerosing cholangitis	6 (23.1)
Hepatitis C virus	5 (19.2)
Hepatocellular carcinoma	3 (11.5)
Autoimmune hepatitis	3 (11.5)
Hepatitis B virus	1 (3.8)
Non-alcohol steatohepatitis	1 (3.8)

Note: Continuous data are presented as median (IQR), and categorical data as numbers (percentage). Abbreviations: dHOPE, dual hypothermic oxygenated machine perfusion; ALT, alanine aminotransferase; AST, aspartate aminotransferase; OLTx, orthotopic liver transplantation; INR, international normalized ratio.

**Table 2:** Donor and liver characteristics.

Donor Characteristics	dHOPE (n = 26)
Age in years (range)	53 (40–60)
Height (cm)	178 (170–180)
Bodyweight (kg)	84 (77–90)
Body mass index (kg/m^2^)	26 (25–29)
Na^+^ (mmol/L)	155 (150–160)
ALT (U/L)	53 (34–100)
AST (U/L)	66 (40–80)
Bilirubin (mg/dL)	0.48 (0.30–0.71)
INR	1.3 (1.1–1.4)
Donor Risk Index ^a^	1.7 (1.4–2.0)
Histological steatosis assessment—n (%) ^b^	
<30% steatosis	25 (96.2)
>30% steatosis	1 (3.8)
Liver weight (g)	1800 (1565–1845)

Note: Continuous data are presented as median (IQR), and categorical data as numbers (percentage). Abbreviations: dHOPE, dual hypothermic oxygenated machine perfusion; ALT, alanine aminotransferase; AST, aspartate aminotransferase; INR, international normalized ratio. ^a^ Donor risk index as described by Feng et al. [8]. ^b^ The steatosis includes large droplets steatosis assessment performed post-transplant.

**Table 3:** dHOPE and transplantation details.

Characteristics	dHOPE (n = 26)
Machine perfusion time (min)	120 (120–180)
Mean portal vein flow (mL/min)	284 (168–394)
Mean hepatic artery flow (mL/min)	95 (78–124)
Anhepatic time (min) ^a^	122 (93–154)
Total OLTx time (min)	435 (360–491)
Intraoperative transfusion of PRBCs (units)	4.00 (0.75–6.00)
Total hypothermic time (min) ^b^	608 (560–687)
CIT (min) ^c^	450 (420–550)
WIT (min) ^d^	65 (50–80)
Post-reperfusion syndrome—n (%) ^e^	6 (23.1)

Note: Continuous data are presented as median (IQR), and categorical data as numbers (percentage). Abbreviations: dHOPE, dual hypothermic oxygenated machine perfusion; CIT, cold ischemic time; WIT, warm ischemic time; OLTx, orthotopic liver transplantation; PRBC, packed red blood cells. ^a^ Anhepatic time is defined as the period from recipient portal vein clamp to liver graft reperfusion. ^b^ Total hypothermic time is defined as the period from the donor’s liver cross-clamping to the removal of the organ from cold storage solution. ^c^ CIT is defined as the period from the donor’s liver cross-clamping to hypothermic machine reperfusion. ^d^ WIT is defined as the period from the removal of the organ from cold storage solution until reperfusion. ^e^ Post-reperfusion syndrome as described by Aggarwal et al. [35].

**Table 4:** Donor and liver graft characteristics in relation to perfusate lactate concentration.

Donor Characteristics	Overall (n = 25)	Lac < 3.45 mmol/L (n = 16)	Lac ≥ 3.45 mmol/L (n = 9)	*p* Value ^a^
Age in years (range)	52 (38–59)	51 (36–56)	56 (35–64)	0.427
Height (cm)	177 (167–180)	175 (166–180)	178 (165–184)	0.834
Bodyweight (kg)	83 (77–90)	80 (71–87)	90 (81–91)	0.122
Body mass index (kg/m^2^)	26 (25–29)	26 (24–29)	26 (25–31)	0.318
Na^+^ (mmol/L)	154 (150–161)	156 (149–170)	150 (148–160)	0.477
ALT (U/L)	47 (34–103)	55 (34–111)	40 (33–88)	0.627
AST (U/L)	66 (40–84)	70 (47–85)	50 (36–101)	0.380
Bilirubin (mg/dL)	0.50 (0.32–0.73)	0.50 (0.41–0.74)	0.30 (0.20–1.0)	0.182
INR	1.3 (1.1–1.4)	1.2 (1.1–1.5)	1.3 (1.0–1.4)	0.771
Donor Risk Index ^b^	1.7 (1.4–1.9)	1.6 (1.4–1.9)	1.8 (1.5–2.0)	0.569
Histological steatosis—n (%) ^c^				0.530
>30% steatosis	1 (4)	0 (0)	1 (11)	
Histological IRI—n (%) ^d^				
Nil	9 (36)	5 (31)	4 (44)	0.671
Mild	10 (40)	7 (44)	3 (33)	0.691
Moderate	5 (20)	3 (19)	2 (22)	0.999
Severe	1 (4)	1 (6)	0 (0)	0.999
CIT (min) ^e^	450 (420–533)	450 (358–477)	516 (435–569)	0.109
Liver weight (g)	1800 (1565–1845)	1737 (1400–1811)	1843 (1800–2350)	0.008

Note: Continuous data are presented as median (IQR), and categorical data as numbers (percentage). Abbreviations: Lac, perfusate lactate concentration measured after 120 min of machine perfusion; ALT, alanine aminotransferase; AST, aspartate aminotransferase; INR, International Normalized Ratio; IRI, ischemia/reperfusion injury; CIT, cold ischemic time. ^a^ Groups compared by Mann-Whitney test for continuous variables and Fisher’s test for categorical variables; Lac ≥ 3.45 mmol/L vs. Lac < 3.45 mmol/L. ^b^ Donor risk index as described by Feng et al. [8]. ^c^ The steatosis includes large droplets steatosis assessment performed post-transplant. ^d^ The severity of IRI was assessed post-transplant, as described by Ali et al. [38]. ^e^ CIT is defined as the period from the donor’s liver cross-clamping to hypothermic machine reperfusion.

**Table 5:** Donor and liver characteristics in relation to EAD occurrence.

Donor Characteristics	Overall (n = 26)	EAD “−” (n = 19)	EAD “+” (n = 7)	*p* Value ^a^
Age in years (range)	53 (40–60)	55 (40–60)	48 (24–67)	0.877
Height (cm)	178 (170–180)	172 (164–180)	180 (170–183)	0.484
Bodyweight (kg)	84 (77–90)	80 (75–90)	85 (81–92)	0.353
Body mass index (kg/m^2^)	26 (25–29)	27 (24–30)	25 (25–29)	0.766
Na^+^ (mmol/L)	155 (150–160)	156 (149–160)	150 (150–162)	0.810
ALT (U/L)	53 (34–100)	70 (40–105)	34 (31–59)	0.139
AST (U/L)	66 (40–80)	68 (40–88)	50 (31–72)	0.311
Bilirubin (mg/dL)	0.48 (0.30–0.71)	0.46 (0.33–0.70)	0.50 (0.20–1.40)	0.944
INR	1.3 (1.1–1.4)	1.2 (1.1–1.5)	1.3 (0.99–1.3)	0.810
Donor Risk Index ^b^	1.7 (1.4–2.0)	1.7 (1.4–2.0)	1.8 (1.4–2.1)	0.855
Histological steatosis assessment—n (%) ^c^				0.269
>30% steatosis	1 (4)	0 (0)	1 (14)	
CIT (min) ^d^	450 (420–550)	450 (380–480)	516 (450–570)	0.041
Liver weight (g)	1800 (1565–1845)	1789 (1439–1813)	1837 (1773–1976)	0.119

Note: Continuous data are presented as median (IQR), and categorical data as numbers (percentage). Abbreviations: EAD, early allograft dysfunction; ALT, alanine aminotransferase; AST, aspartate aminotransferase; INR, International Normalized Ratio; CIT, cold ischemic time. ^a^ Groups compared by Mann-Whitney test for continuous variables and Fisher’s test for categorical variables; EAD “+” vs. EAD “−”. ^b^ Donor risk index as described by Feng et al. [8]. ^c^ The steatosis includes large droplets steatosis assessment performed post-transplant. ^d^ CIT is defined as the period from the donor’s liver cross-clamping to hypothermic machine reperfusion.

## Data Availability

The datasets used and/or analyzed during the current study are available from the corresponding author upon reasonable request.

## Supplementary Materials

The following supporting information can be downloaded at: https://www.mdpi.com/article/10.3390/jcm14020471/s1, Figure S1. Overview of ROC analysis outcomes for perfusate FMN measurements at different time points concerning EAD predictability. Figure S2. Overview of ROC analysis outcomes for perfusate lactate measurements at different time points concerning EAD predictability. Figure S3. Overview of ROC analysis outcomes for DRI, total hypothermic time, and CIT concerning EAD predictability. Figure S4. Correlation between perfusate FMN measurements after 30 min of perfusion and lactate levels after 120 min. Figure S5. Overview of clinically relevant outcomes in patients with EAD. Figure S6. Relationship between liver graft weight and perfusate lactate concentrations after 120 min of perfusion Table S1. Recipient characteristics in relation to perfusate lactate concentration. Table S2. Recipient characteristics in relation to EAD occurrence.
