# Supplementary material for: Real-Time Biomarkers of Liver Graft Quality in Hypothermic Oxygenated Machine Perfusion
Source: J Clin Med. 2025 Jan 13;14(2):471. doi: 10.3390/jcm14020471 (PMC11766178; doi:10.3390/jcm14020471)
Supplement: Supplementary file 1 [file jcm-14-00471-s001.zip › jcm-3348666-supplementary.pdf]

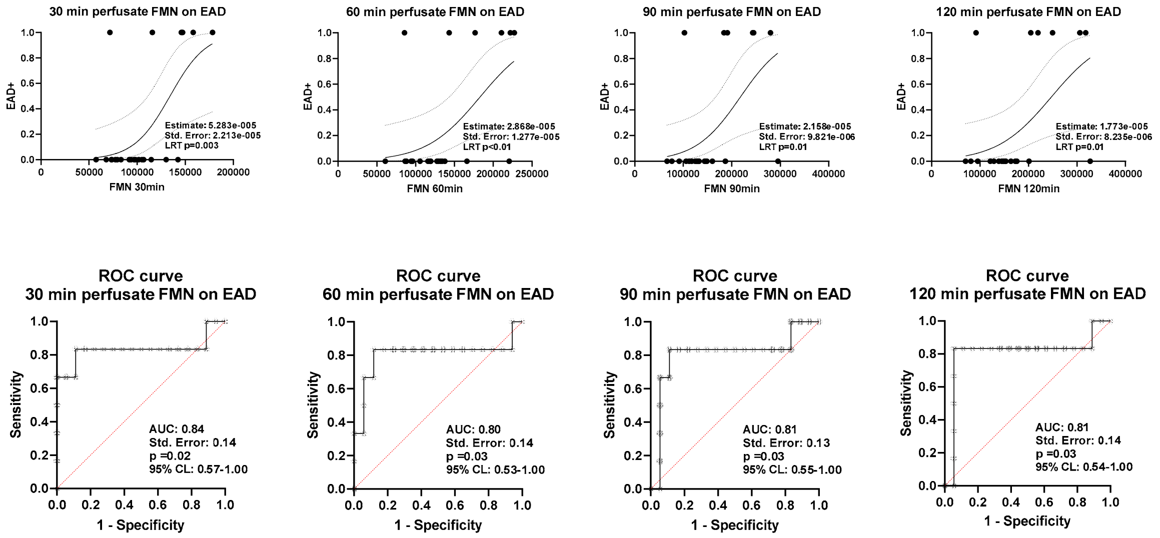

Figure S1. Overview of ROC analysis outcomes for perfusate FMN measurements at different timepoints concerning EAD predictability.

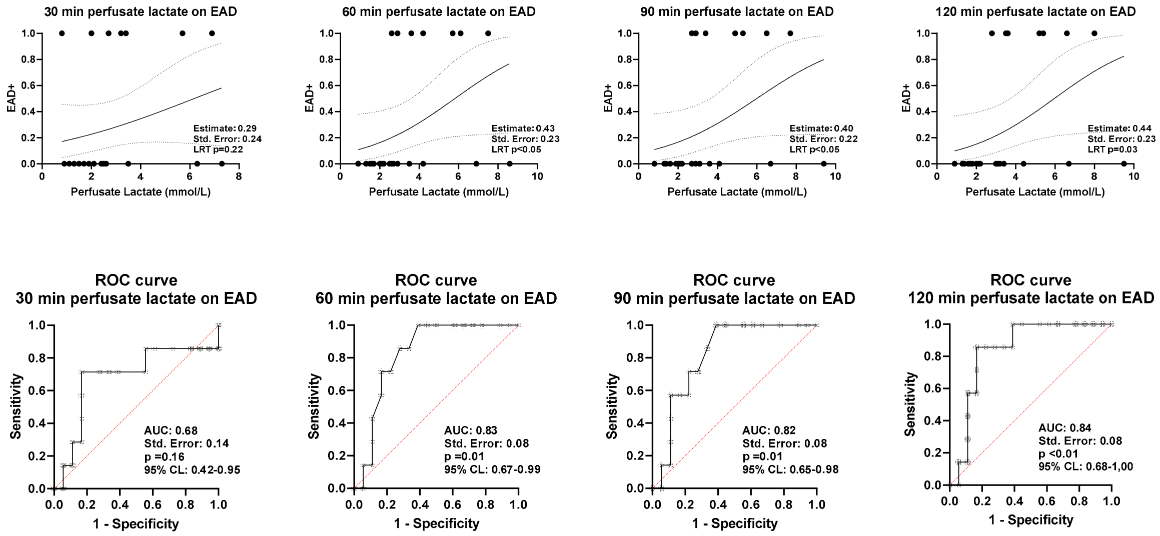

Figure S2. Overview of ROC analysis outcomes for perfusate lactate measurements at different timepoints concerning EAD predictability.

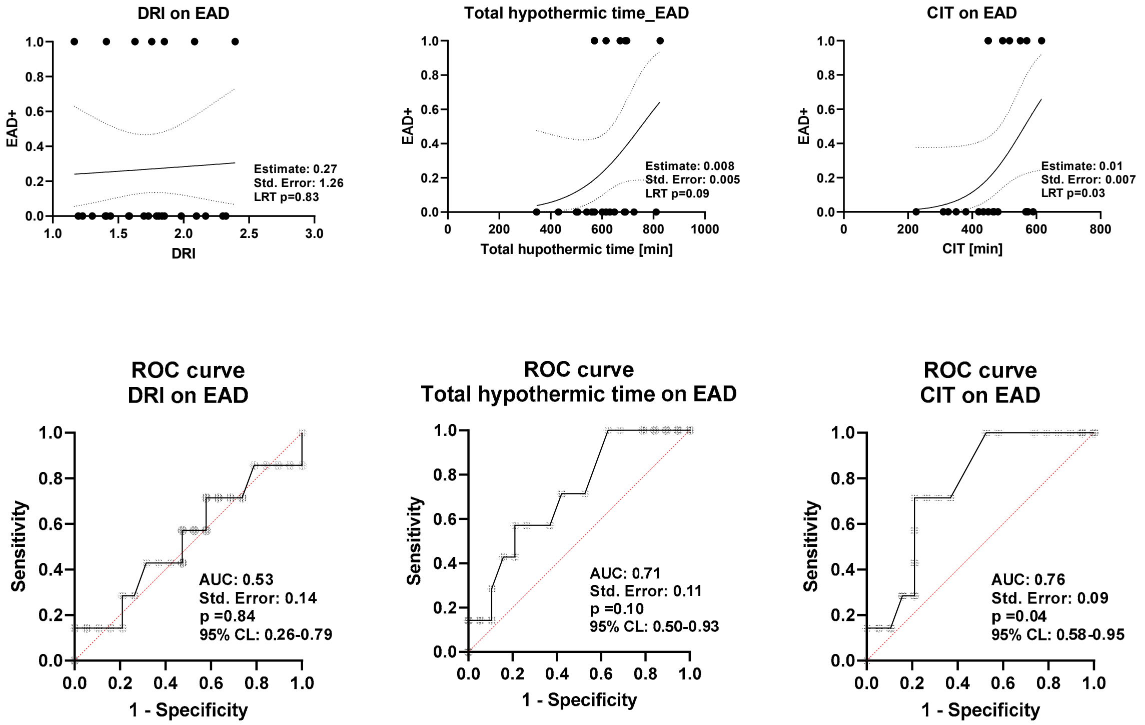

Figure S3. Overview of ROC analysis outcomes for DRI, total hypothermic time and CIT concerning EAD predictability.

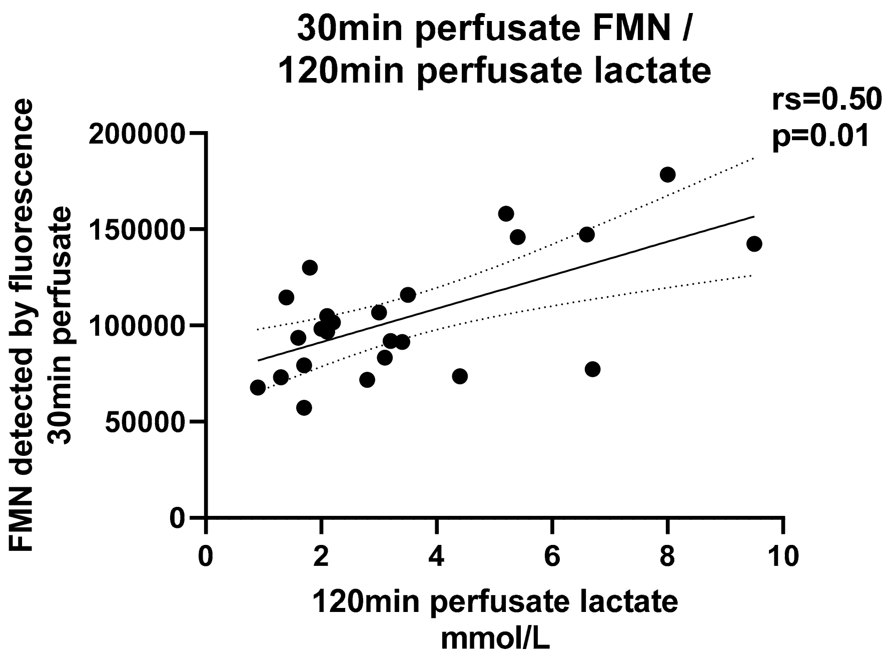

Figure S4. Correlation between perfusate FMN measurements after 30 minutes of perfusion and lactate levels after 120 minutes.

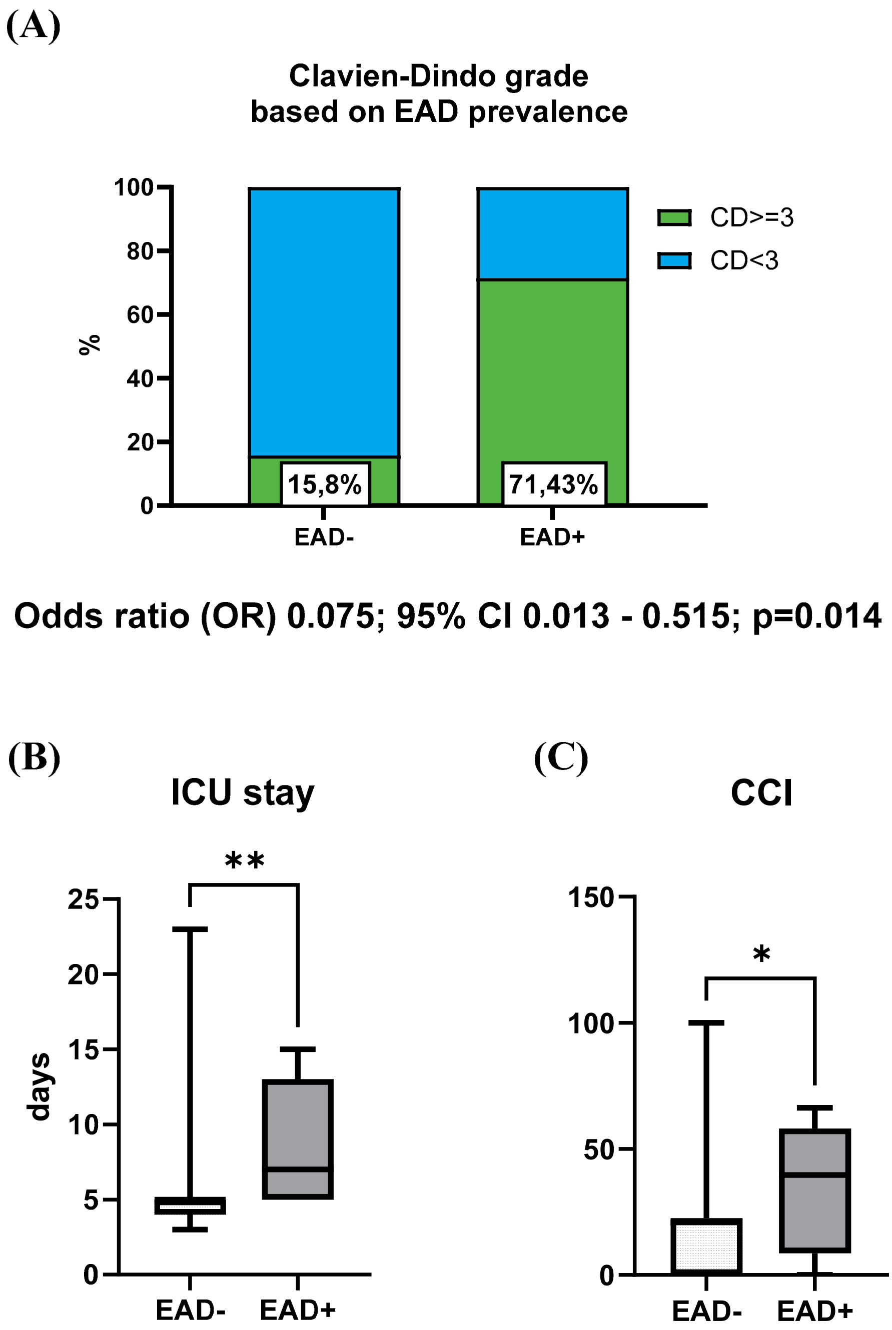

Figure S5. Overview of clinically relevant outcomes in patients with EAD.

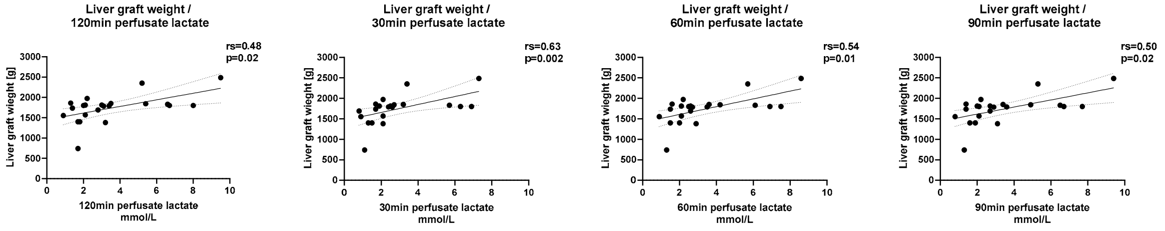

Figure S6. Relationship between liver graft weight and perfusate lactate concentrations after 120 minutes of perfusion.

Table S1. Recipient characteristics in relation to perfusate lactate concentration.

Recipient characteristics	Overall (n=25)	Lac <3.45 mmol/L (n=16)	Lac=>3.45 mmol/L (n=9)	p value Lac => vs <3.45 mmol/L[a]
Age in years (range)	47 (40-63)	46 (37-60)	55 (41-69)	0.295
Sex - n (%)				0.394
Female	8 (32)	4 (25)	4 (44)	
Male	17 (68)	12 (75)	5 (56)	
Body mass index (kg/m^2)	26 (23-28)	25 (22-27)	27 (24-33)	0.136
Model for end-stage liver disease score	11 (8-21)	12 (8-21)	11 (8-18)	0.966
ALT at OLTx (IU/mL)	61 (21-139)	68 (22-154)	55 (20-111)	0.495
AST at OLTx (IU/mL)	56 (32-116)	61 (31-152)	41 (32-84)	0.923
Albumin at OLTx (g/dL)	3.8 (3.4-4.6)	3.7 (3.4-4.3)	4.2 (3.5-4.9)	0.139
Bilirubin at OLTx (mg/dL)	1.5 (0.8-8.6)	1.9 (0.8-8.8)	1.3 (0.9-6.1)	0.708
INR	1.1 (1.0-1.3)	1.1 (1.0-1.4)	1.3 (1.0-1.4)	0.989
Creatinine (mg/dL)	0.96 (0.73-1.20)	0.91 (0.68-1.20)	0.99 (0.86-1.30)	0.395
Transplant indication – n (%)				
Alcohol-related liver disease	6 (24)	3 (19)	3 (33)	0.630
Non-alcohol steatohepatitis	1 (4)	0 (0)	1 (11)	0.360
Hepatitis C virus	5 (20)	1 (6)	4 (44)	0.040
Primary biliary cirrhosis	1 (4)	0 (0)	1 (11)	0.360
Primary sclerosing cholangitis	2 (8)	2 (13)	0 (0)	0.520
Autoimmune hepatitis	2 (8)	2 (13)	0 (0)	0.520
Hepatocellular carcinoma	5 (20)	4 (25)	1 (11)	0.621

Note: Continuous data are presented as median (IQR), categorical data as numbers (percentage).

Abbreviations: Lac, perfusate lactate concentration measured after 120 minutes of machine perfusion; ALT, alanine aminotransferase; AST, aspartate aminotransferase; INR, International Normalized Ratio; OLTx, orthotopic liver transplantation.

[a]Groups compared by Mann-Whitney test for continuous variables and Fisher's test for categorical variables.

Table S2. Recipient characteristics in relation to EAD occurrence.

Recipient characteristics	Overall (n=26)	EAD "-" (n-19)	EAD "+" (n=7)	p value EAD"+" vs "-" [a]
Age in years (range)	46 (38-62)	47 (36-62)	43 (39-67)	0.745
Sex - n (%)				>0.999
Female	8 (31)	6 (32)	2 (29)	
Male	18 (69)	13 (68)	5 (71)	
Body mass index (kg/m^2)	26 (23-28)	26 (23-27)	28 (23-35)	0.254
Model for end-stage liver disease score	12 (8-21)	11 (10-21)	12 (8-20)	0.944
ALT at OLTx (IU/mL)	63 (21-132)	55 (18-154)	98 (22-124)	0.418
AST at OLTx (IU/mL)	61 (32-111)	55 (30-125)	76 (41-88)	0.357
Albumin at OLTx (g/dL)	3.9 (3.5-4.7)	3.8 (3.5-4.4)	4.8 (3.2-5.0)	0.139
Bilirubin at OLTx (mg/dL)	1.6 (0.8-9.3)	1.8 (0.8-9.0)	1.5 (0.7-10.0)	0.684
INR	1.2 (1.0-1.3)	1.1 (1.0-1.3)	1.3 (1.0-1.5)	0.815
Creatinine (mg/dL)	0.94 (0.73-1.20)	0.91 (0.71-1.30)	0.98 (0.81-1.20)	0.662
Transplant indication – n (%)				
Alcohol-related liver disease	6 (23)	4 (21)	2 (29)	>0.999
Non-alcohol steatohepatitis	1 (4)	0 (0)	1 (14)	0.269
Hepatitis C virus	5 (19)	3 (16)	2 (29)	0.588
Primary biliary cirrhosis	1 (3.8)	0 (0)	1 (14)	0.269
Primary sclerosing cholangitis	6 (23)	6 (32)	0 (0)	0.146
Autoimmune hepatitis	3 (12)	3 (16)	0 (0)	0.540
Hepatocellular carcinoma	5 (19)	3 (16)	2 (29)	0.588

Note: Continuous data are presented as median (IQR), categorical data as numbers (percentage).

Abbreviations: Lac, perfusate lactate concentration measured after 120 minutes of machine perfusion; ALT, alanine aminotransferase; AST, aspartate aminotransferase; INR, International Normalized Ratio; OLTx, orthotopic liver transplantation.

[a]Groups compared by Mann-Whitney test for continuous variables and Fisher's test for categorical variables.
